# Treatment with Creatine Monohydrate in Spinal and Bulbar Muscular Atrophy: Protocol for a Randomized, Double-Blind, Placebo-Controlled Trial

**DOI:** 10.2196/resprot.8655

**Published:** 2018-03-05

**Authors:** Yasuhiro Hijikata, Masahisa Katsuno, Keisuke Suzuki, Atsushi Hashizume, Amane Araki, Shinichiro Yamada, Tomonori Inagaki, Daisuke Ito, Akihiro Hirakawa, Fumie Kinoshita, Masahiko Gosho, Gen Sobue

**Affiliations:** ^1^ Department of Neurology Nagoya University Graduate School of Medicine Nagoya Japan; ^2^ Innovation Center for Clinical Research National Center for Geriatnics and Gerontology Obu Japan; ^3^ Biostatistics Section Center for Advanced Medicine and Clinical Research Nagoya University Graduate School of Medicine Nagoya Japan; ^4^ Department of Clinical Trial and Clinical Epidemiology Faculty of Medicine University of Tsukuba Tsukuba Japan; ^5^ Research Division of Dementia and Neurodegenerative Disease Nagoya University Graduate School of Medicine Nagoya Japan

**Keywords:** spinal and bulbar muscular atrophy, creatine, randomized controlled trials

## Abstract

**Background:**

Although spinal and bulbar muscular atrophy (SBMA) has been classified as a motor neuron disease, several reports have indicated the primary involvement of skeletal muscle in the pathogenesis of this devastating disease. Recent studies reported decreased intramuscular creatine levels in skeletal muscles in both patients with SBMA and transgenic mouse models of SBMA, which appears to contribute to muscle weakness.

**Objective:**

The present study aimed to examine the efficacy and safety of oral creatine supplementation to improve motor function in patients with SBMA.

**Methods:**

A randomized, double-blind, placebo-controlled, three-armed clinical trial was conducted to assess the safety and efficacy of creatine therapy in patients with SBMA. Patients with SBMA eligible for this study were assigned randomly in a 1:1:1 ratio to each group of placebo, 10 g, or 15 g daily dose of creatine monohydrate in a double-blind fashion. Participants took creatine or placebo orally 3 times a day for 8 weeks. Outcome measurements were results of neurological assessments, examinations, and questionnaires collected at baseline and at weeks 4, 8, and 16 after a washout period. The primary endpoint was the change in handgrip strength values from baseline to week 8. The secondary endpoints included the following: results of maximum voluntary isometric contraction tests of extremities; tongue pressure; results of the 15-foot timed walk test and the rise from bed test; modified quantitative myasthenia gravis score; respiratory function test results; activities of daily living assessed with the Revised Amyotrophic Lateral Sclerosis Functional Rating Scale and the Spinal and Bulbar Muscular Atrophy Functional Rating Scale; skeletal muscle mass measured with dual-energy X-ray absorptiometry; urinary 8-hydroxydeoxyguanosine levels; and questionnaires examining the quality of life, swallowing function, and fatigue.

**Results:**

Participant enrollment in the trial started from June 2014 and follow-up was completed in July 2015. The study is currently being analyzed.

**Conclusions:**

This is the first clinical trial evaluating creatine therapy in SBMA. Given that creatine serves as an energy source in skeletal muscles, recovery of intramuscular creatine concentration is expected to improve muscle strength.

**Trial Registration:**

University Hospital Medical Information Network Clinical Trials Registry UMIN000012503; https://upload.umin.ac.jp/cgi-open-bin/ctr_e/ctr_view.cgi?recptno=R000014611 (Archived by WebCite at http://www.webcitation.org/6xOlbPkg3).

## Introduction

Spinal and bulbar muscular atrophy (SBMA), also known as Kennedy’s disease, is an adult-onset, X-linked neuromuscular disease characterized by limb, trunk, and facial weakness [1-3]. Most of the patients with SBMA experience finger tremor or muscle cramp before the onset of muscular weakness. In general, the progression of neurological dysfunction is slow, with the average interval between the onset of muscular weakness and death being approximately 20 years [4]. Patients at a terminal stage of SBMA are certainly bound to be in a wheelchair or bedridden state, and some of these patients develop recurrent aspiration pneumonia due to bulbar palsy [4]. SBMA is caused by the expansion of a CAG trinucleotide repeat, encoding a polyglutamine tract, within the first exon of the androgen receptor (AR) gene [5]. The ligand-dependent accumulation of the pathogenic AR proteins in the nucleus is fundamental to the molecular pathogenesis of this disease, providing a potential target for therapy development [6-8].

Although motor neurons are the primary target of polyglutamine-mediated toxicity, several studies have implied skeletal muscle involvement in SBMA pathogenesis. Serum levels of creatine kinase are higher in patients with SBMA than in those with amyotrophic lateral sclerosis (ALS) [4,9,10]. Patients with SBMA demonstrated both neurogenic and myopathic features in the muscle biopsy [11]. Moreover, it has been demonstrated that skeletal muscle pathology preceded neurodegeneration in knock-in and transgenic mouse models of SBMA [12-15]. In skeletal muscles, the polyglutamine-expanded AR induces transcriptional alterations of several genes that are implicated in muscle function [12,16]. Recent studies showed transcription alterations in skeletal muscle energy metabolism that are a consequence of mutant AR expression in SBMA muscle [17,18]. These findings imply a direct involvement of the skeletal muscle in SBMA pathogenesis [19].

We previously identified the serum creatinine level as a sensitive serological biomarker for motor dysfunction in patients with SBMA [20]. Serum creatinine is produced from creatine, which is mostly present in skeletal muscle tissues. Serum creatinine levels are, therefore, construed as an index of skeletal muscle mass. However, we recently reported that serum creatinine levels in patients with SBMA are markedly decreased due to the decreased muscular uptake of creatine resulting from the pathogenic AR-mediated downregulation of SLC6A8, a creatine transporter [21]. In addition, both animal and clinical studies indicated glycolytic-to-oxidative fiber type switch in the skeletal muscle of SBMA [17,18,22], which may also contribute to the decreased intramuscular creatine in SBMA, given that type 1 slow-twitch fibers have lower phosphocreatine contents compared with type 2 fast-twitch fibers [23].

Creatine is converted to creatine phosphate by creatine kinase and exists as a storage material of high energy phosphate in skeletal muscle [24]. It has been reported that orally ingested creatine increases the amount of creatine phosphate in the skeletal muscle [25]. Intramuscular creatine phosphate functions as an energy source when energy demand is increased by rapid movement. Creatine also regulates intramuscular calcium homeostasis and mitochondrial ADP-stimulated respiration in both slow- and fast-twitch fibers [26,27].

Based on these findings, we hypothesized that supplementation of creatine will attenuate muscle weakness in patients with SBMA. Hence, we designed the CREatine Complemental medication for Kennedy’s disease in Eight weeks Trial (the CRECKET study), a randomized controlled trial (RCT) that examines the efficacy and safety of creatine therapy in patients with SBMA. Although the efficacy of creatine replacement therapy has been demonstrated in certain muscular diseases such as Duchenne muscular dystrophy [25,28,29], its effectiveness in SBMA has yet to be validated. This study is the first attempt to evaluate the efficacy and safety of creatine supplementation in patients with SBMA.

## Methods

### Ethical Approval and Trial Registration

This study was conducted in compliance with the Helsinki Declaration and approved by the Ethics Committee of Nagoya University Graduate School of Medicine. The study was registered with the University Hospital Medical Information Network clinical trials registry (UMIN000012503) before the start of the recruitment period.

### Study Design

**Figure 1:**
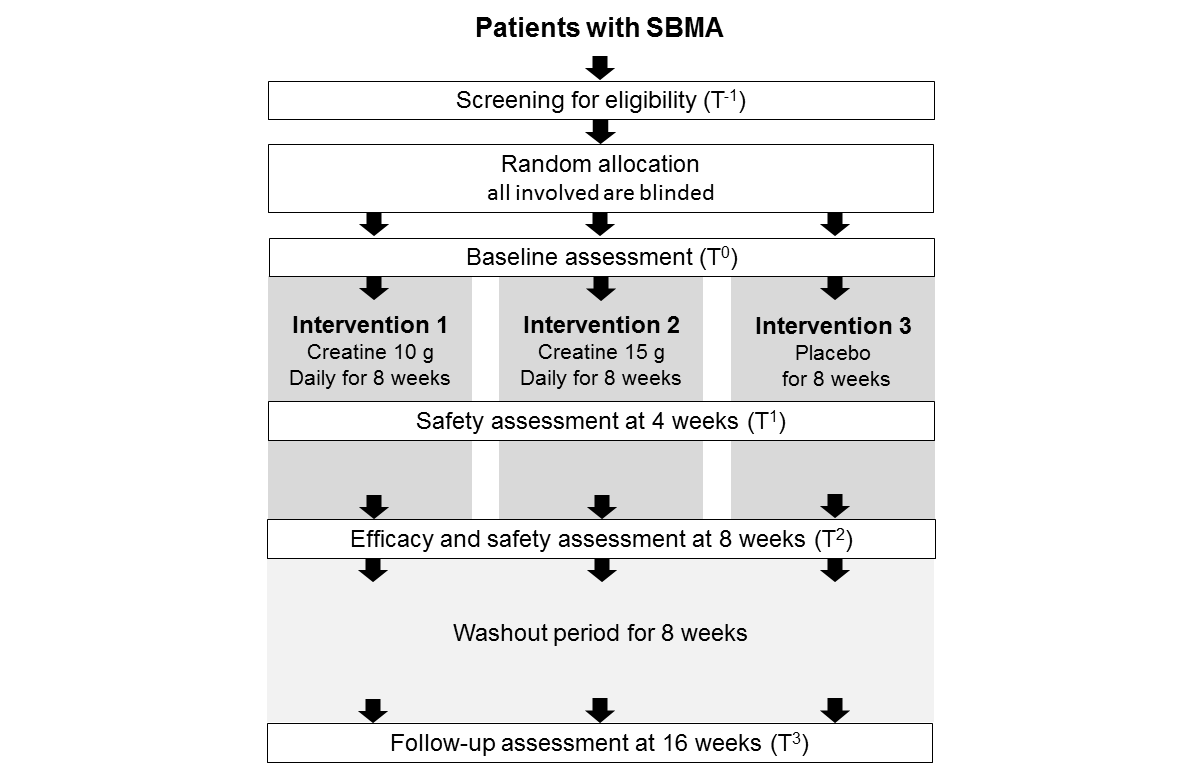
Flow chart of design and enrollment procedures. SBMA: spinal and bulbar muscular atrophy.

This study is a randomized, double-blind, placebo-controlled, three-armed, phase II trial to assess the safety and efficacy of creatine monohydrate in patients with SBMA in accordance with the Consolidated Standards of Reporting Trials (CONSORT) guideline. After obtaining informed consent, a primary assessment (T^-1^) of the potential participants was performed, whereby participants were screened for eligibility within 4 weeks prior to the start of the study medication by measuring their blood parameter values and serum testosterone levels. Patients with SBMA eligible for this study were allocated randomly in a 1:1:1 ratio to receive placebo, 10 g daily of creatine monohydrate (SAVAS; Meiji Co, Ltd, Tokyo, Japan), or 15 g daily of creatine in a double-blinded fashion. The duration of the intervention was 8 weeks. The study participants underwent four assessments (T^0^, T^1^, T^2^, and T^3^) composed of neurological assessments, examinations, and questionnaires (Figure 1 and Table 1). The study intervention was initiated after baseline assessment (T^0^). For the purpose of safety assessment and compliance, participants were assessed again after 4±1 treatment weeks (T^1^). Efficacy assessments were scheduled after 8±1 treatment weeks (T^2^). Follow-up assessments were performed after the 8-week washout period (T^3^). All assessments were performed at Nagoya University Hospital.

### Intervention

Participants took creatine or placebo orally after every meal, 3 times a day, for 8 weeks. In the high-dose creatine group, participants took 5 g of creatine monohydrate powder daily after every meal (total 15 g). In the low-dose creatine group, participants took powder medicine containing 3.33 g of creatine monohydrate powder and 1.67 g of lactose daily after every meal (total 10 g). In the placebo group, powder medicine containing 5 g of lactose was taken daily after each meal (total 15 g). Participants started the oral administration within 3 days after the baseline assessment (T^0^). Participants were instructed to pay attention to the following points when taking the medicine: (i) To take powder medicine suspended in sufficient water or juice; (ii) take the powder medicine always after meals, and not before meals; (iii) avoid warm water to suspend powder medicine; and (iv) take all the medicine including the precipitated powder.

To avoid the influence of other treatments on the evaluation of the effectiveness of creatine, participants were prohibited to use the following medicines until the end of the evaluation period; luteinizing hormone-releasing hormone (LH-RH) agonists, LH-RH antagonists, testosterone drugs, 5-alpha-reductase inhibitors, anti-androgen drugs, protein anabolic hormone, progesterone drugs, estrogen drugs, unapproved drugs, and creatine supplements. Participants were also prohibited to start rehabilitation of extremities or undergo castration until the end of the evaluation.

### Participants

Patients with SBMA were eligible for participating in the study. All participants were recruited from the Department of Neurology at Nagoya University according to the inclusion and exclusion criteria. The inclusion criteria were as follows: (1) Male patients who present one or more of the following motor symptoms: (i) muscle weakness of extremities, (ii) muscle atrophy of extremities, and (iii) bulbar palsy; (2) Patients whose genetic testing results showed that they bear at least 38 CAG repeats within the AR gene; (3) Patients who were twenty (≥ 20) to eighty (< 80) years of age at the time of informed consent; (4) Patients who can visit the hospital regularly as outpatients; (5) Patients whose renal function meets the following criteria: creatinine at < 1.5 × Upper Limit of Institutional Reference Value; (6) Patients who provided written informed consent by themselves.

**Table 1:** Study schedule and assessments.

Item		Prestudy		Double-blind, placebo-controlled study			Washout	Early termination
		Screening (T^-1^)	Registration	T^0^ (0 week)	T^1^ (4 weeks)	T^2^ (8 weeks)	T^3^ (16 weeks)	
**Enrollment**								
	Written consent	✓						
	Patient characteristics	✓						
	Screening tests	✓						
	Registration		✓					
**Assessment**								
	Handgrip strength			✓	✓	✓	✓	✓
	Quantitative muscle test			✓	✓	✓	✓	✓
	Timed walk test			✓	✓	✓	✓	✓
	Rise from bed test			✓	✓	✓	✓	✓
	Tongue pressure			✓	✓	✓	✓	✓
	Modified QMG score^a^			✓	✓	✓	✓	✓
	Dual-energy X-ray absorptiometry			✓		✓	✓	✓
	Respiratory function test			✓		✓		✓
	ALSFRS-R^b^ (Japanese version)			✓		✓		✓
	SBMAFRS^c^			✓		✓		✓
	SDQ^d^ (Japanese version)			✓		✓		✓
	SWAL-QOL^e^ (Japanese version)			✓		✓		✓
	ALSAQ-5^f^ (Japanese version)			✓		✓		✓
	MFI-20^g^ (Japanese version)			✓		✓		✓
	Urinary 8-OHdG^h^			✓		✓	✓	✓
	Serum creatine/creatinine			✓	✓	✓	✓	✓
	Urinary creatine/creatinine			✓		✓		✓
	Subjective/objective concomitant symptoms			✓	✓	✓	✓	✓
**Laboratory test**								
	Blood test			✓	✓	✓	✓	✓
	Biochemical test	✓		✓	✓	✓	✓	✓
	Urine test			✓	✓	✓	✓	✓
	Blood pressure, body weight			✓	✓	✓	✓	✓
	Serum testosterone	✓						
	Genetic test^i^ (CAG repeat length)			✓				

^a^QMG score: Quantitative Myasthenia Gravis score

^b^ALSFRS-R: Revised Amyotrophic Lateral Sclerosis Functional Rating Scale

^c^SBMAFRS, Spinal and Bulbar Muscular Atrophy Functional Rating Scale

^d^SDQ: Swallowing Disturbance Questionnaire

^e^SWAL-QOL: Swallowing Quality of Life Questionnaire

^f^ALSAQ: Amyotrophic Lateral Sclerosis Assessment Questionnaire

^g^MFI: Multidimensional Fatigue Inventory

^h^8-OHdG: 8-hydroxydeoxyguanosine

^i^Re-examination under the same conditions.

Patients who met any of the following criteria were excluded from the study: (1) Patients who have taken LH-RH agonists, LH-RH antagonists, testosterone drugs, anti-androgen drugs, estrogen drugs, or unapproved drugs within 48 weeks and 5-alpha-reductase inhibitors within 24 weeks before agreement acquisition; (2) Patients whose serum testosterone level is below the lower limit of normal; (3) Patients who have taken creatine supplementation within 8 weeks before agreement acquisition; (4) Patients who have severe complications, such as malignancy, heart failure, and renal failure; (5) Patients who were determined ineligible for this study by the investigator or coinvestigators.

### Recruitment and Settings

Neurologists, ie, investigator or coinvestigators, identified patients with SBMA and provided them with sufficient information about the explanatory statement and the informed consent form before their participation. All participants gave their written informed consent for trial participation prior to the screening. After the screening assessment for eligibility, neurologists evaluated the eligibility of the patients using a checklist and register patients for this trial.

### Randomization and Blinding

Randomization was performed centrally with the use of an online system (Waritsuke-kun; Mebix, Inc, Tokyo, Japan). Dynamic random allocation was done with minimization on the basis of the patients’ disease duration (0–9 or ≥ 10 years from onset) and past history of LH-RH agonist treatment to reduce the bias. A double-blind study was conducted to achieve a higher standard of scientific rigor in evaluating the efficacy and safety of creatine. A biostatistician from an external facility (MG) was delegated to the “Allocator” who was responsible for treatment allocation. The Allocator ensured that it was impossible to determine whether the study agent was creatine monohydrate or placebo by its appearance or package and randomly allocated patients to the study agents in a repeatable manner. The Allocator ensured that the allocation code/list has been kept in a sealed envelope before opening it, and that the allocation has been concealed before unblinding. During the study period, serum creatine, serum creatinine, urinary creatine, and urinary creatinine were measured by a clinical laboratory measuring institution (LSI Medience Corporation, Tokyo, Japan) to maintain the blindness in this study. The results of the examination were kept at the clinical laboratory measuring institution without disclosure. After unblinding, the examination results of these items were reported to the investigator.

### Outcome Measures

The primary endpoint of this trial was the change in handgrip strength values from baseline to week 8 (T^2^). Handgrip strength values were measured using an electronic hand dynamometer. For measurement, the patients were instructed to keep their elbows at an angle of 90°, their forearms in a neutral rotation, and their wrists not flexed or pronated. The grip power was measured twice on each side, and the average of the maximal power of both sides was recorded. Secondary outcome measures included muscle strength measured by tongue pressure [30], maximum voluntary isometric contraction (bilateral shoulder flexors and extensors, elbow flexors and extensors, knee flexors and extensors, and ankle flexors and extensors), five components of the Quantitative Myasthenia Gravis score (excluding the ptosis and diplopia sections) [31], 15-foot timed-walk test [32], and rise-from-bed test, which measures the time in changing the position from the supine position on the bed to the sitting position. We also measured changes in the Revised Amyotrophic Lateral Sclerosis Functional Rating Scale (ALSFRS-R) [7,33,34] and the Spinal and Bulbar Muscular Atrophy Functional Rating Scale, which is a validated, disease-specific scale with a high sensitivity to disease progression in SBMA [35]. We evaluated muscle mass with dual-energy X-ray absorptiometry (DXA) using the fan-beam technology (Discovery A; Hologic Inc, Bedford, MA). The sum of the appendicular lean soft tissue mass measured with DXA has been validated by the measurement of the skeletal muscle mass using magnetic resonance imaging and computed tomography [36-38]. As other outcome measures, we analyzed the subjective assessment of swallowing function (Swallowing Disturbance Questionnaire and Swallowing Quality of Life Questionnaire) [39,40], respiratory function values (vital capacity, forced vital capacity [FVC], forced expiratory volume one second percent, peak expiratory flow, and V50/V25), 5-item Amyotrophic Lateral Sclerosis Assessment Questionnaire [41], the Multidimensional Fatigue Inventory [42], and urinary 8-hydroxydeoxyguanosine (8-OHdG) as a marker of oxidative stress [43].

### Efficacy, Safety, and Tolerability Data Analyses

#### Efficacy Data Analyses

The primary endpoint of the efficacy analysis was the change in grip strength at 8 weeks of administration from baseline. In the primary analysis, we examined the superiority of 10 g/day and 15 g/day of creatine administration groups over the placebo-treated group using the Dunnett’s multiple comparison test with the placebo treated group as the control. In the secondary analysis, we also estimated a dose-response relationship among the 3 groups using the linear contrast test. The mixed model for repeated measures and random slope mixed effect model were also applied. All the analyses were conducted based on the intent to treat principle that included all randomly assigned patients who received the study medication and provided at least one postbaseline efficacy datum, as well as the per-protocol set that included all intent to treat patients with no important protocol violations relevant to assessing the study agent efficacy. A two-sided *P*<0.05 was considered to be statistically significant. All statistical analyses were performed using SAS (version 9.4; SAS Institute Inc, Cary, NC, USA).

#### Safety/Tolerability Data

Safety was evaluated in all patients who received the study agent. In each patient, safety and tolerability assessments including vital signs, medical examination findings, clinical laboratory data, and intensity of adverse events (AEs) were evaluated. Severe AEs were defined as incapacitating or causing inability to work or undertake usual activities. Each AE was coded to a preferred term and associated organ system according to an established and validated adverse reaction dictionary (MedDRA/J, version 18.0); AEs were monitored by an independent data and safety monitoring board.

### Sample Size Calculation

In this study, we intended to enroll 45 patients with SBMA. This was based on previous studies that examined the efficacy of creatine therapy for muscular dystrophy. In those placebo-controlled trials, intergroup differences of the change from baseline were 6.4 to 16.2% (SD 19.8 to 30.7%) in the maximum voluntary muscle strength [25,28,29,44]. The pharmacological mechanism of increasing intramuscular creatine concentration is common in SBMA and muscular dystrophy. In addition, in our preliminary examination, patients with SBMA displayed a lower intramuscular creatine concentration compared with disease controls including muscular dystrophy, suggesting that a larger creatine efficacy may be expected in SBMA. Based on the above, the number of required participants was calculated assuming that the intergroup difference of the change rates of the muscle strength is 28.0% and the SD is 20.0%.

## Results

All 45 participants have been enrolled starting in June 2014 and follow-up was completed in July 2015. The study is currently being analyzed.

## Discussion

This trial is the first randomized control trial evaluating the efficacy and safety of creatine monohydrate in patients with SBMA. Currently, there is no treatment available for counteracting muscle weakness in patients with SBMA. Restoration of the muscle creatine concentration may be a candidate therapeutic strategy for SBMA.

To be eligible for randomization, we set the patients’ disease duration from the onset of muscle weakness as the allocation factor because SBMA is a slow progressive disease and clinical symptoms worsen with the disease progression. In addition, since prior LH-RH agonist administration may influence the evaluation of the efficacy, we set the past treatment history of LH-RH agonists as the other allocation factor.

We used the handgrip strength as a primary endpoint. Muscle weakness in patients with SBMA is known to stem from the cytotoxicity of mutant AR in both motor neurons and skeletal muscles. In SBMA, handgrip strength decreases gradually over the course of the disease progression [20]. Furthermore, in a randomized, double-blind comparative study of creatine therapy for Duchenne muscular dystrophy, the grip strength was reported to improve significantly compared with the placebo group [29]. In the Cochrane Collaborative Plan systematic review of creatine therapy for muscular diseases, creatine therapy has been shown to be effective for muscle strengthening when using quantitative strength measurements including hand grip strength as the primary endpoint [45]. Therefore, we chose the handgrip strength as the primary endpoint in our trial. In the previous clinical trials of creatine therapy, other outcome measures such as quantitative muscle testing of extremities, pulmonary function testing, body composition measured by DXA, subjective assessment of improvement of muscle weakness, and urinary 8-OHdG as a marker of oxidative stress to DNA were adopted [28,29,44,46]. In our trials, we added five components of the Quantitative Myasthenia Gravis score as a secondary outcome to evaluate muscular endurance.

Although it was suggested that creatine may have a neuroprotective effect in ALS animal model [47], it was reported that creatine did not have a statistically significant effect on survival, ALSFRS-R progression, or percent predicted FVC progression in the Cochrane Collaborative Plan systematic review (although creatine 5 to 10 g per day was well-tolerated with no serious adverse events in all studies [48]).

In a systematic review of creatine therapy for muscle disorders with meta-analysis of 14 RCTs [45], the median creatine administration period was 8 weeks (3-6 months). Since the pharmacological mechanism anticipated for creatine therapy in SBMA is similar to that in other muscle disorders, the administration period in this study was also set at 8 weeks.

## Abbreviations

8-OHdG: 8-hydroxydeoxyguanosine
AE: adverse event
ALS: amyotrophic lateral sclerosis
ALSFRS-R: the Revised Amyotrophic Lateral Sclerosis Functional Rating Scale
AR: androgen receptor
DXA: dual-energy X-ray absorptiometry
FVC: forced vital capacity
LH-RH: luteinizing hormone-releasing hormone
RCT: randomized controlled trial
SBMA: spinal and bulbar muscular atrophy
